# Comparison of different insulin resistance surrogates in predicting hyperuricemia in non-diabetic Yi residents in Yunnan Province

**DOI:** 10.3389/fendo.2025.1523771

**Published:** 2025-12-15

**Authors:** Mengyao Dao, Xianyu He, Yanmei Ji, Ni Guo, Wenjun Li, Qing Xiong, Ni Meng, Dan Zhou, Ting Pi, Xiaofeng Zong, Meirong Yang, Xinyi Liu, Zhongjuan Wang, Xingfang Jin

**Affiliations:** 1 Clinical Medical Research Center for Cardiovascular Disease of Yunnan Province, Yan’an Hospital Affiliated to Kunming Medical University, Kunming, Yunnan, China; 2 Department of Cardiovascular Surgery, Yan’an Hospital Affiliated to Kunming Medical University, Kunming, Yunnan, China; 3 Department of Endocrinology, Yan’an Hospital Affiliated to Kunming Medical University, Kunming, Yunnan, China; 4 Department of Pharmacy, Yan’an Hospital Affiliated to Kunming Medical University, Kunming, Yunnan, China

**Keywords:** insulin resistance surrogate, hyperuricemia, non-diabetic population, Yi resident in Yunnan, predictive ability

## Abstract

**Background and purpose:**

Hyperuricemia (HUA) is one of the most common metabolic diseases in China, with an incidence of up to 36% across different ethnic groups. Insulin resistance (IR) plays a specific role in the occurrence and development of HUA, but relevant studies on the non-diabetic Yi residents in Yunnan are limited. Our study aimed to explore the triglyceride-glucose index (TyG), triglyceride-glucose and body mass index (TyG-BMI), triglyceride/high-density lipoprotein cholesterol ratio (TG/HDL-C), and the metabolic score for insulin resistance (METS-IR) among non-diabetic Yi residents in Yunnan Province, and identify the IR surrogates with high predictive ability for HUA.

**Materials and methods:**

From November 2018 to September 2023, our group finally included 765 Yi residents aged ≥18 years on the basis of the inclusion criteria. The participants were divided into the non-HUA and HUA groups. Logistic regression analysis was used to describe the association between four IR surrogates and HUA and calculate odds ratios (ORs), while the receiver operating characteristic curve was utilized to assess the prediction ability of these four IR surrogates.

**Results:**

Among the 765 non-diabetic Yi residents in Yunnan, 666 were in the non-HUA group, and 99 were in the HUA group. A history of smoking, alcohol consumption, hypertension, and hyperlipidemia, body mass index (BMI) ≥ 24 kg/m^2^, triglyceride, serum creatinine levels, TyG, TyG-BMI, TG/HDL-C, and METS-IR were found positively correlated with the occurrence of HUA (*P* < 0.05). In contrast, in female, HDL-C levels were negatively correlated with the occurrence of HUA (*P* < 0.05). Logistic regression analysis showed that after adjusting for age, smoking history, alcohol consumption history, history of hyperlipidemia, history of hypertension, systolic and diastolic blood pressures, cholesterol, low-density lipoprotein, serum creatine levels, TyG, TyG-BMI, TG/HDL-C, and METS-IR were found independent influencing factors for the occurrence of HUA. Receiver operating characteristic curve analysis showed that these four IR surrogates had certain predictive ability for HUA.

**Conclusion:**

This study found that in non-diabetic Yi residents in six areas of Yunnan Province, the TyG, TyG-BMI, TG/HDL-C, and METS-IR were positively correlated with HUA. These four IR surrogates were good at predicting HUA.

## 1 Introduction

Insulin resistance (IR) refers to the weakened/diminished biologic response of target tissues (primarily the liver, skeletal muscle, and adipose tissue) to insulin stimulation. As a result, the pancreatic β-cells augment their insulin secretory capacity to offset the IR resulting in the increase in plasma insulin concentration (hyperinsulinemia) such that glucose tolerance/level remains normal or only slightly impaired (1). Thus, non-diabetic individual can be insulin resistant. All tissues expressing insulin receptors can become insulin resistant, IR produces different effects in different organs and tissues. In central nervous system (CNS), IR causes obesity because appetite is controlled by the action of insulin in the CNS and neuronal insulin signaling is required for both body weight control and glucose homeostasis. Acute administration of insulin directly into the CNS was shown to suppress hepatic glucose output (2). Hepatic IR generates postprandial hyperglycemia in mice lacking insulin receptor substrate 1(Irs1) fed with high-fat diet (3). Insulin potentiates the β-cell insulin secretory response to glucose in healthy, insulin-sensitive persons (4). Skeletal muscles store glucose by converting it into glycogen and triglycerides. IR in skeletal muscles of muscle-specific insulin receptor deficient mice was shown to have altered fat metabolism (elevated fat mass, serum triglycerides, and free fatty acids) associated with type 2 diabetes (5) though other tissues also play significant roles in insulin-regulated glucose disposal. The heart is an insulin-responsive and energy-consuming organ that requires a constant fuel supply to maintain intracellular ATP levels for myocardial contraction. Both heart-specific IRS1 and IRS2 double-knockout mice and liver-specific IRS1 and IRS2 double-knockout mice prove that cardiac IR promotes heart failure (6). IR in vascular endothelium promotes hypertension by not prouducing enough potenet vasodilator NO and disrupts glucose homeostasis by not promoting glucose disposal in skeletal muscle and adipose tissue and not inhibiting gluconeogenesis in liver (7). IR in osteoblast, a bone-specific cell, impairs whole-body glucose homeostasis by enhancing osteocalcin activity through inhibition of osteocalcin carboxylation and promoting glucose metabolism (8). People having sedentary lifestyle, a lack of physical activity, with excess body fat, especially around their belly, a family history of insulin resistance or even with normal body weight can be insulin resistant but may not have diabetes because they secrete more insulin than usual to manage blood sugar levels before diagnosed with prediabetes (9, 10). IR/hyperinsulinemia plays major patho-physiological role in type 2 diabetes, obesity, hypertension, coronary artery disease, dyslipidemias, and a cluster of metabolic and cardiovascular abnormalities known as metabolic syndrome (MS) (1). IR is often associated with central obesity, hypertension, dyslipidemia, atherosclerosis and polycystic ovarian syndrome, this constellation of symptoms is often referred to as syndrome X, or insulin resistance syndrome (11).

IR impairs glucose uptake by the cells of tissues and its catabolism, resulting in a compensatory increase in insulin production from hyper-responsive pancreatic islet β-cell causing the state of hyperinsulinemia (12, 13). IR is present in the patients with impaired glucose tolerance (IGT) or non-insulin-dependent diabetes mellitus (NIDDM) of nonobese individuals with normal oral glucose tolerance (14). The state of hyperinsulinemia/IR is now considered as the root causative factor for metabolic syndrome (MS), non-alcoholic fatty liver disease (NAFLD), polycystic ovary syndrome (PCOS), obesity-related type 2 diabetes (T2D) and atherosclerotic cardiovascular disease (ASCVD) (13, 14). IR/hyperinsulinemia can enhance very-low-density lipoprotein (VLDL) synthesis, leading to hypertriglyceridemia (1)and Hyperuricemia (HUA) (15). Reports indicate HUA prevalence of 10.7%-45% in African T2DM populations, where it exacerbates diabetic complications (16), and 28.1% in Japanese T2DM cohorts (17). HUA is an independent risk factor for the onset of diabetes through the promotion of inflammatory responses and oxidative stress, thereby impairing insulin sensitivity. IR precedes the onset of diabetes and is closely associated with HUA. Early detection and intervention of HUA are therefore warranted for the clinical relevance for both diabetic and non-diabetic populations (18, 19).These findings underscore the urgent need for a convenient, cost-effective screening tool to enable early identification and intervention in high-risk populations with IR.

Insulin resistance (IR) refers to the decreased/weakened response of target tissues to insulin that causes disorders of glucose and lipid metabolism (20). The current “gold standard” for evaluating IR is the hyperinsulinemic-euglycemic clamp (HEC) test. Other markers to evaluate IR include the homeostasis model assessment of insulin resistance (HOMA-IR), frequently sampled intravenous glucose tolerance test (FSIGT), oral glucose tests, and the insulin sensitivity index. However, these tests are expensive, complicated, and time-consuming. Moreover, they are often difficult to perform in clinical settings, especially in primary health institutions with insufficient medical resources, and may only be suitable for scientific research at this stage (20). Therefore, many recent studies have proposed the use of the triglyceride-glucose index (TyG) (21), triglyceride-glucose and body mass index (TyG-BMI) (22), triglyceride/high-density lipoprotein cholesterol ratio (TG/HDL-C) (23), and the metabolic score for detection of IR (METS-IR) (24). These four non-insulin-based IR surrogates have also been shown to be closely correlated with IR (21–23, 25). These four surrogate markers require non-insulin-based biochemical tests of fasting blood samples combined with anthropometric measurements readily available in primary care to allow rapid and easy assessments of patients’ IR status. The TyG, TyG-BMI, TG/HDL-C, and METS-IR are closely related to HUA, and are independent influencing factors for the occurrence of HUA. A cross-sectional study conducted in China revealed that all four IR surrogates (TyG, TyG-BMI, TG/HDL-C, and METS-IR) could predict the risk of HUA in middle-aged and elderly individuals with type 2 diabetes mellitus, among them, the METS-IR index demonstrated superior predictive value, particularly in females, showing a significantly higher area under the curve (AUC) compared to males (26). Similarly, a large-scale study in a non-diabetic population in the United States confirmed that these four IR surrogates were significantly associated with an increased risk of HUA (27). Furthermore, a cross-sectional study of an Iranian population with coronary artery disease indicated that the TyG index and TyG-BMI exhibited the highest predictive ability for HUA in these patients (28). Domestic and foreign studies have demonstrated that all four markers can assess the risk of HUA, regardless of whether there is diabetes or not (26–28). Therefore, we selected these four IR surrogates for our study.

According to the Yunnan Statistical Yearbook of 2023 (28), the Yunnan Province contains numerous ethnic minorities. These ethnic minorities account for 33.12% of the total population of Yunnan Province, of which the Yi people account for a substantially large proportion (10.74% of the total population). The Yi people also differ from other ethnic groups in terms of living environment, diet culture, education level, and physical activity (29). The occurrence of HUA has been reported to differ in relation to race, geographical location, sex, age, lifestyle, and eating habits (31–35). Among non-diabetic Yi resident in Yunnan, the relationships between the above-mentioned four IR surrogates and the prevalence of HUA remain unclear. Thus, we examined the potential efficacy of these IR surrogates as predictors of HUA in this specific demographic.

In this study we aimed to explore correlation between these IR four surrogates and HUA among non-diabetic Yi residents in Yunnan and compare their diagnostic significance and feasibility to identify a reliable, rapid, and simple prediction tool that can be used in clinical practice, especially in primary health institutions with insufficient medical resources with the hope that the findings will provide a scientific basis for early prediction and scientific prevention of HUA among non-diabetic Yi residents.

## 2 Materials and methods

### 2.1 Study population

**Figure 1:**
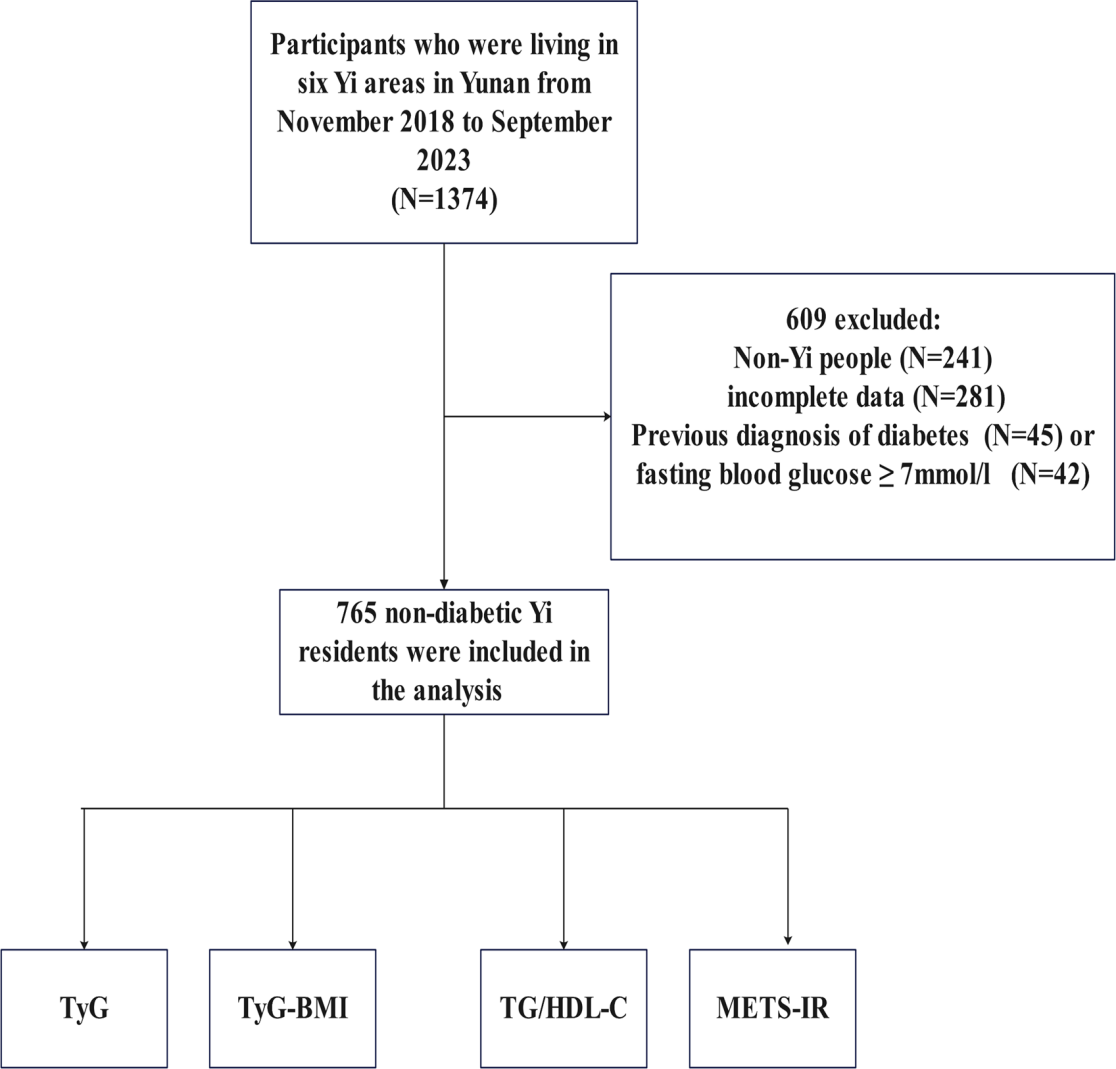
Flowchart of the study. HUA, hyperuricemia; TyG, triglyceride glucose; TyG-BMI, triglyceride glucose with body mass index; TG/HDL-C, the ratio of triglycerides divided by high-density lipoprotein cholesterol; METS-IR, the metabolic score for insulin resistance.

A simple random sampling technique was employed to select participants. From November 2018 to September 2023, a total of 1374 participants were recruited using this method in six areas of Yunnan province. The inclusion criteria were as follows: (1) age ≥ 18 years; (2) completed the questionnaire survey, anthropometric assessments, and laboratory tests with no missing data. The exclusion criteria were as follows: (1) incomplete data; (2) Han and other ethnicities; (3) diabetes diagnosed by a doctor (“yes” to the statement “the doctor told you that you have diabetes”) or fasting plasma glucose (FPG) level ≥ 7.0 mmol/L in this test.Among them, 609 were excluded for incomplete data(N = 281), non-Yi people(N = 241), previous diagnosis(N = 45) or fasting blood glucose ≥ 7.0 mmol/L(N = 42), thus, 765 Yi residents aged ≥18 years were eventually included. **Figure 1** shows the participant enrollment flowchart. The formulas for the indicators:(1)BMI (kg/m^2^)= Weight (kg)/Height (m)^2^; (2)TyG= ln[TG (mg/dL) × FPG (mg/dL)/2]; (3)TyG-BMI= TyG × BMI; (4)TG/HDL-C= TG (mg/dL)/HDL-C (mg/dL); (5) METS-IR= ln[(2*FPG (mg/dL) + TG (mg/dL)]*BMI/ln (HDL-C (mg/dL)). The formulas for conversion of values between mg/dL and mmol/L or μmol/L: 1 mg/dL FPG = 18 mmol/L FPG, 1 mg/dL TG = 88.5 mmol/L TG, and 1 mg/dL HDL-C = 38.67 mmol/L HDL-C.

Sample size was determined based on the following formula for simple random sampling:


n=Za22P×(1−P)δ2×deff

n: Sample size; *Z*: Statistical value. At a significance level (*α*) of 0.05 and a 95% confidence interval, *Z_α/2_* = 1.96; *P*: Prevalence rate of HUA. Based on published literature, the overall prevalence rate among Chinese residents is 13.3% (1). *δ*: Allowable error. Considering the feasibility of resources, time, and manpower in this study, *δ* was set to 11%. *deff*: Design effect. To account for regional variations in the prevalence of HUA, *deff* was set to 1.5.

The minimum sample size for each region was calculated as n=55 based on the statistical formula. To account for potential data loss and invalid responses at a rate of 5%–10%, a minimum of 61 participants per township was targeted. Accordingly, the total minimum sample size across all six townships was determined to be 366. Ultimately, our study enrolled 765 participants, significantly exceeding the calculated minimum requirement.

### 2.2 Diagnostic criteria and data collection

#### 2.2.1 Diagnostic criteria

Uric acid concentration in human blood samples was detected by an ultraviolet method (35), and SU levels > 428 μmol/L in males and >357 μmol/L in females were used as the diagnostic criterion for HUA (36). In 1997, the World Health Organization (WHO) (37) defined a smoker as a person who had smoked continuously or cumulatively for six months or more over a lifetime. We used the same definition to identify participants with a smoking history in this study. Similarly, individuals who consumed alcohol at least once a week were considered to have a history of alcohol consumption (38). Hypertension was identified if the patients showed SBP ≥ 140 mmHg and/or DBP ≥ 90 mmHg on three blood pressure measurements conducted in the office on different days in the absence of antihypertensive drugs or if patients with a history of hypertension were currently taking antihypertensive drugs, even if their blood pressure is lower than 140/90 mmHg (39). For hyperlipidemia, patients were diagnosed as showing dyslipidemia when they showed one or more of the following abnormalities on the basis of fasting venous serum detection markers (40): hypercholesterolemia (total CHOL level ≥ 5.2 mmol/L), hypertriglyceridemia (TG level ≥ 1.7 mmol/L), and high low-density lipoprotein cholesterolemia (LDL-C level ≥ 3.4 mmol/L), low high-density lipoprotein cholesterolemia (HDL-C level < 1.0 mmol/L). Moreover, on the basis of the age groups in developing countries defined by the WHO, participants were categorized as young (18–44 years), middle-aged (45–59 years), young-old (60–74 years), and old people (≥75 years).

#### 2.2.2 Questionnaire

This study utilized a questionnaire-based methodology, with all surveys administered face-to-face by a single trained investigator. The survey content included the participants’ age, sex, smoking history, alcohol consumption history, hypertension history, hyperlipidemia history.

#### 2.2.3 Anthropometric measurements

Standard measurement protocols were employed, CSTF-5000 height and weight instrument was used to measure participants’ height and weight, with BMI subsequently calculated. Blood pressure was measured using an electronic sphygmomanometer. Before measurement, participants were instructed to sit quietly and rest for 5–10 minutes. The purpose of the procedure was explained, and their cooperation was obtained to ensure a relaxed state, minimizing anxiety or tension. Measurements were taken with the participant in a seated position, with the right upper arm fully exposed and resting on a table at heart level. Blood pressure was measured twice, with a 1-minute interval between measurements. The average of the two readings was recorded as the final blood pressure value.

#### 2.2.4 Laboratory indicator measurement

Participants were required to fast for 8 hours overnight before the blood draw. Venous blood samples were collected and sent to KingMed Diagnostics for analysis. The following parameters were evaluated: Fasting plasma glucose (FPG) level, high-density lipoprotein cholesterol (HDL-C) level, low-density lipoprotein cholesterol (LDL-C) level, triglyceride (TG) level, serum creatinine (Scr) level, serum urate (SU) level, total cholesterol (CHOL) level, and other data.

### 2.3 Statistical analysis

IBM SPSS Statistics for Windows, version 25.0 (IBM Corp, Armonk, N.Y, USA) was used for analysis. The normality test typically employs the Kolmogorov-Smirnov test (for large sample sizes) and the Shapiro-Wilk test (for small sample sizes, usually n ≤ 50). Additionally, visual inspections through Q-Q plots (Quantile-Quantile plots) and histograms are commonly used to assist in assessing the distribution. Continuous data were expressed as *X ± S* if they conformed to a normal distribution and as *M* (*P*_25_, *P*_75_) for non-normally distributed data. Categorical variables were presented as numbers in percentages (%). For measurement data meeting the assumptions of normality and homogeneity of variance, the Student’s t-test was used between the HUA and non-HUA groups; for data not meeting these assumptions, the Kruskal–Wallis test was employed; for categorical data, the Chi-square test or Fisher’s exact probability test was applied. Spearman correlation analysis was used for correlation analysis. Logistic regression analysis was used to calculate the odds ratios (ORs) and 95% confidence interval (CIs) of TyG, TyG-BMI, TG/HDL-C, and METS-IR to evaluate the association between these four IR surrogates and HUA. The area under the receiver operating characteristic curve (ROC) was used to measure the predictive ability of different IR surrogates for HUA. The area under the ROC curve (AUC) values exceeds 0.5, a value closer to 1 indicates superior predictive performance. The cutoff value for the indices was determined on the basis of the highest Youden index in the ROC curves. A *p*-value < 0.05 was considered statistically significant.

## 3 Results

### 3.1 Baseline characteristics

**Figure 2:**
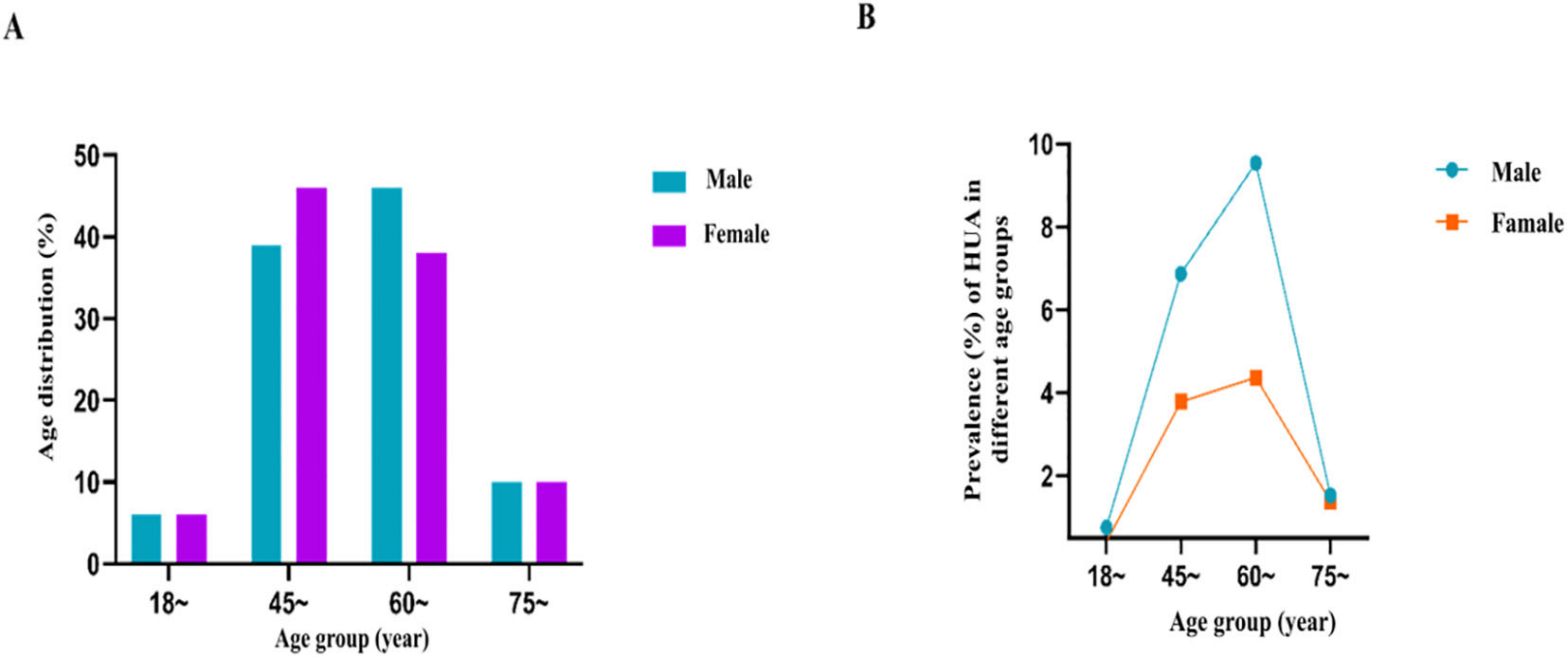
**(A)** Age distribution of different genders; **(B)** Prevalence of HUA in different gender groups.

**Figure 3:**
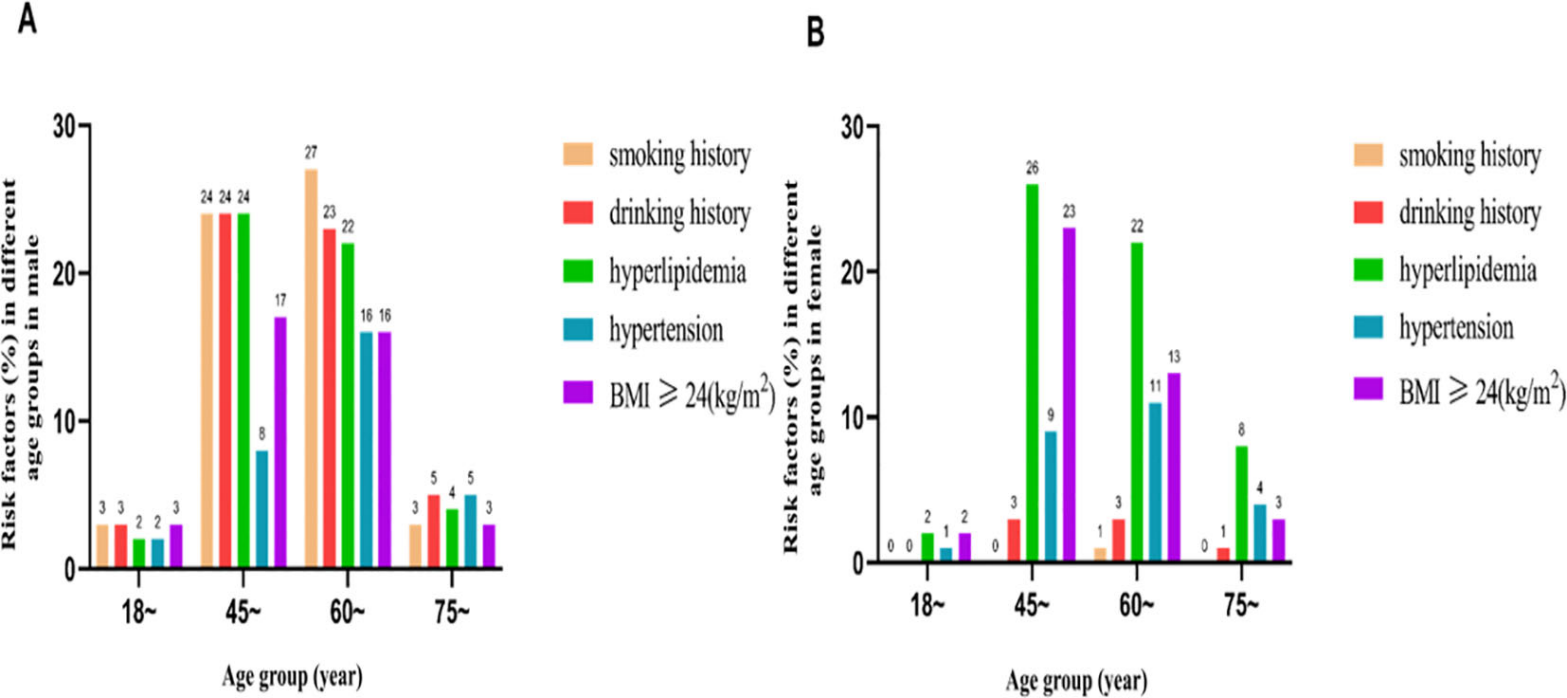
**(A)** The rate of risk factors in different age groups(male). **(B)** The rate of risk factors in different age groups(female).

A total of 765 Yi residents (262 males and 503 females) aged ≥18 years were included in the study. The prevalence of HUA was 12.94% (99/765) overall, 18.70% (49/262) in males, and 9.94% (50/503) in females. The study population included 15 (6%), 101 (39%), 121 (46%), and 25 (10%) males aged 18–44 years, 45–59 years, 60–74 years, and ≥75 years, respectively; the corresponding values for female participants were 29 (6%), 233 (46%), 189 (38%), and 52 (10%), respectively. As seen in **Figure 2A**, the majority of participants were between 45 and 74 years old. In this age range, the prevalence of HUA was higher in men than in women (**Figure 2B**). The detection rates of the risk factors (smoking, alcohol consumption, hyperlipidemia, hypertension, and overweight/obesity) by age group are shown in **Figure 3**.

Compared to the non-HUA group, participants with HUA had significantly higher levels of TG, FPG, Scr, SU, BMI, SBP, DBP, TyG, TyG-BMI, TG/HDL-C, and METS-IR (all *p* < 0.05). In addition, a higher proportion of men, smokers, alcohol consumers, and subjects with hypertension or hyperlipidemia were observed. In contrast, the HUA group had lower HDL-C levels and a lower proportion of women (*p* < 0.05) (**Table 1**).

**Table 1:** Baseline characteristics of subjects.

Variables	The Yi nationality (n=765)			
	Without HUA (n=666)	With HUA (n=99)	Ζ/χ^2^/t	*P*-value
Age (years)	60.01 ± 10.60	61.76 ± 10.91	-1.524	0.128
Gender (n, %) Male Female	213 (31.98%) 453 (68.02%)	49 (49.49%) 50 (50.51%)	11.739	**0.001**
Smoking history (n, %)			4.876	0.027
Yes	125 (18.77%)	28 (28.28%)		
No	541 (81.23%)	71 (71.72%)		
Drinking history (n, %)			6.317	0.012
Yes	140 (21.02%)	32 (32.32%)		
No	526 (78.98%)	67 (67.68%)		
Hyperlipidemia (n, %)			9.210	0.002
Yes	356 (53.45%)	69 (69.70%)		
No	310 (46.55%)	30 (30.30%)		
Hypertension (n, %)			21.596	<0.001
Yes	156 (23.42%)	45 (45.45%)		
No	510 (76.58%)	54 (54.55%)		
BMI (kg/m^2^)	23.36 ± 3.78	25.22 ± 4.40	-3.997	**<0.001**
CHOL(mmol/L)	5.46 (4.91, 6.24)	5.64 (5.05, 6.25)	-1.143	0.253
TG(mmol/L)	1.35 (0.95, 1.94)	2.07 (1.16, 3.24)	-5.586	**<0.001**
LDL-C(mmol/L)	3.28 (2.67, 3.86)	3.19 (2.69, 3.89)	-0.141	0.888
HDL-C(mmol/L)	1.59 (1.32, 1.85)	1.45 (1.20, 1.72)	-3.382	**0.001**
FPG(mmol/L)	5.05 ± 0.57	5.37 ± 0.65	-5.084	**<0.001**
Scr(μmol/L)	70.15 ± 13.23	80.89 ± 15.95	-6.380	**<0.001**
SU(μmol/L )	281.00 (234.00, 326.00)	444.10 (400.00, 500.00)	-15.440	**<0.001**
SBP (mmHg)	138.53 ± 22.78	149.86 ± 21.39	-4.654	**<0.001**
DBP (mmHg)	83.39 ± 12.79	91.08 ± 14.16	-5.499	**<0.001**
TyG	8.58 (8.25, 8.95)	9.04 (8.55, 9.50)	-6.313	**<0.001**
TyG-BMI	202.10 ± 39.15	230.14 ± 47.72	-5.572	**<0.001**
TG/HDL-C	1.90 (1.24, 3.14)	3.16 (1.76, 5.73)	-5.579	**<0.001**
METS-IR	32.12 (28.01, 36.60)	37.53 (32.21, 43.26)	-5.629	**<0.001**

HUA, hyperuricemia; SU, serum urate; SBP, systolic blood pressure; DBP, diastolic blood pressure; FPG, fasting blood glucose level; HDL-C, high-density lipoprotein cholesterol level; LDL-C, low-density lipoprotein cholesterol; TG, triglyceride level; Scr, serum creatinine; CHOL, total cholesterol; BMI, body mass index; TG/HDL-C triglyceride/high-density lipoprotein cholesterol ratio; TyG, triglyceride-glucose index; TyG-BMI, triglyceride-glucose and body mass index; METS-IR metabolic score for insulin resistance.

Bold values indicates that P is less than 0.05.

### 3.2 Correlation analysis

Analysis of correlation results showed that smoking history, alcohol consumption history, hypertension history, hyperlipidemia history, BMI ≥ 24 kg/m^2^, TG and Scr levels, TyG, TyG-BMI, TG/HDL-C, and METS-IR were all positively correlated with the occurrence of HUA (*P* < 0.05); in contrast, female sex and HDL-C level were negatively correlated with the occurrence of HUA (*P* < 0.05), as shown in **Table 2**. The strength of correlation increases with the absolute value of the correlation coefficient(*r)*: a coefficient closer to 1 or -1 indicates a stronger correlation, while a value closer to 0 suggests a weaker correlation. Typically, the strength of correlation is interpreted as follows: 0.8 - 1.0: Very strong correlation; 0.6 - 0.8: Strong correlation; 0.4 - 0.6: Moderate correlation; 0.2 - 0.4: Weak correlation; 0.0 - 0.2: Very weak or no correlation.

**Table 2:** Analysis of the correlation between IR surrogates and HUA of Yi nationality.

Variables	*r*-value	*P*-value
Gender	-0.124	0.001
Smoking history	0.080	0.027
Drinking history	0.091	0.012
Hyperlipidemia	0.110	0.002
Hypertension	0.168	<0.001
BMI	0.160	<0.001
BMI≥24(kg/m^2^)	0.167	<0.001
TG	0.202	<0.001
HDL-C	-0.122	0.001
Scr	0.232	<0.001
TyG	0.228	<0.001
TyG-BMI	0.216	<0.001
TG/HDL-C	0.204	<0.001
METS-IR	0.202	<0.001

Only *P*<0.05 are shown. Evaluation:male=0, female=1.

*r*-value: the value of a correlation coefficient is bounded between -1 and +1. Its mathematical sign (positive or negative) reflects the directional nature of the relationship;

(1)0.8 – 1.0: Very strong correlation; (2)0.6 – 0.8: Strong correlation; (3)0.4 – 0.6: Moderate correlation (4)0.2 – 0.4: Weak correlation;

(5)0.0 – 0.2: Very weak or no correlation.

### 3.3 Multivariate logistic regression analysis

Due to the presence of multicollinearity among TyG, TyG-BMI, TG/HDL-C, and METS-IR, separate logistic regression analyses were conducted for each indicator. After adjusting for confounders (Model 1:unadjusted; Model 2:adjusted for age, smoking history, drinking history, hyperlipidemia, hypertension, SBP, and DBP; Model 3:Model 2 + CHOL, LDL-C, Scr) and collinearity diagnosis, three models of IR surrogates were established. The results showed that in the sex subgroup, after adjusting for age, smoking history, alcohol consumption history, history of hyperlipidemia, history of hypertension, SBP, DBP, and CHOL, LDL-C, and Scr levels, the TyG, TyG-BMI, TG/HDL-C, and METS-IR were all independent factors influencing the occurrence of HUA in non-diabetic male and female Yi residents in Yunnan Province (*P* < 0.05), as shown in **Table 3**. Among both male and female Yi populations, these four surrogates of insulin resistance were identified as significant independent influencing factors for HUA.

**Table 3:** ORs and 95% CIs for highest versus the lowest quartiles in logistic regressions predicting the presence of HUA in males/females.

Variables	Model 1	*P*-value	Model 2	*P-*value	Model 3	*P-*value
TyG						
male	3.174 (1.981, 5.086)	**<0.001**	3.142 (1.761, 5.607)	**<0.001**	3.754 (1.891, 7.449)	**<0.001**
female	2.793 (1.703, 4.581)	**<0.001**	2.790 (1.551, 5.016)	**0.001**	2.648 (1.436, 4.882)	**0.002**
TyG-BMI						
male	1.014 (1.007, 1.021)	**<0.001**	1.011 (1.003, 1.020)	**0.010**	1.011 (1.001, 1.020)	**0.024**
female	1.016 (1.010, 1.023)	**<0.001**	1.016 (1.009, 1.024)	**<0.001**	1.017 (1.009, 1.025)	**<0.001**
TG/HDL-C						
male	1.053 (1.004, 1.104)	**0.032**	1.040 (0.999, 1.083)	0.054	1.064 (1.016, 1.115)	**0.009**
female	1.067 (0.997, 1.142)	0.062	1.066 (1.001, 1.136)	**0.048**	1.077 (1.007, 1.152)	**0.031**
METS-IR						
male	1.062 (1.026, 1.101)	**0.001**	1.053 (1.010, 1.097)	**0.015**	1.054 (1.008, 1.103)	**0.022**
female	1.086 (1.049, 1.125)	**<0.001**	1.088 (1.046, 1.131)	**<0.001**	1.090 (1.047, 1.135)	**<0.001**

Values are odds ratio (95%*CI*) derived from multivariable logistic regression models.

Model 1: unadjusted.

Model 2: adjusted for age, smoking history, drinking history, hyperlipidemia, hypertension, SBP, and DBP.

Model 3: Model 2 + CHOL, LDL-C, Scr.

Bold values indicates that P is less than 0.05.

### 3.4 Receiver operating characteristic curve analysis

**Figure 4:**
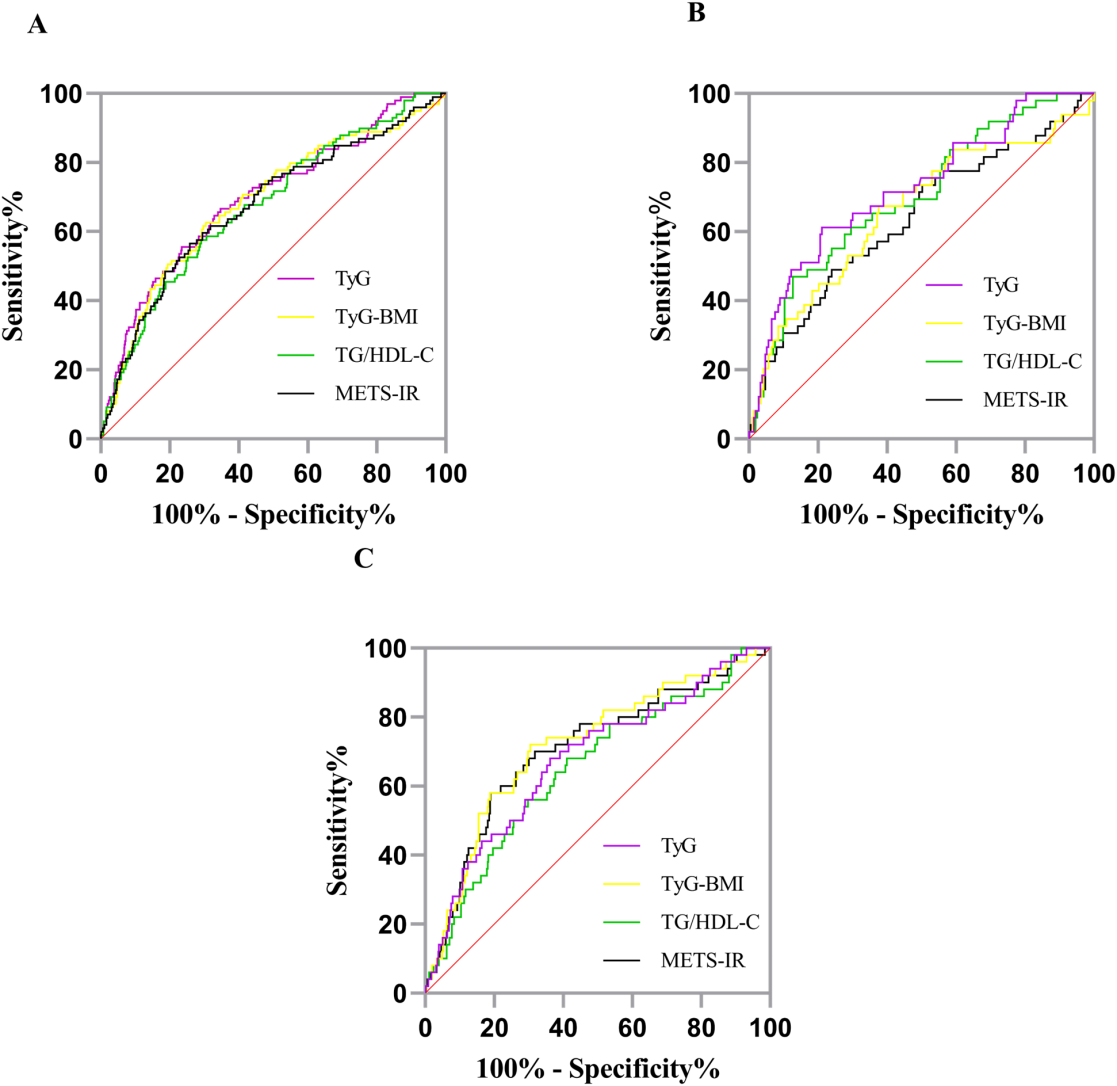
ROC curves for predicting HUA using different IR surrogate markers. **(A)** total; **(B)** male; **(C)** female. HUA, hyperuricemia; TyG, triglyceride-glucose index; TyG-BMI, triglyceride-glucose with body mass index; TG/HDL-C, the ratio of triglyceride level divided by the high-density lipoprotein cholesterol level; METS-IR, the metabolic score for insulin resistance. Sensitivity, probability of HUA detection; 100% - Specificity%, probability of false alarm.

The area under the ROC curve (AUC) values of TyG, TyG-BMI, TG/HDL-C, and METS-IR were all >0.6, indicating that all IR surrogates had predictive ability for the identification of HUA. The AUC of TyG (AUC = 0.696) is closest to 1, indicating superior predictive performance. The optimal cutoff values of TyG, TyG-BMI, TG/HDL-C, and METS-IR were 8.98, 186.52, 0.85, and 32.21, respectively, as shown in **Figure 4A** and **Table 4**. Among the Yi non-diabetic males, the AUC of TyG (0.724) is closest to 1, indicating superior predictive performance, as shown in **Figure 4B** and **Table 4**. Among non-diabetic Yi women, the AUC of TyG-BMI (0.718) is closest to 1, indicating superior predictive performance, as shown in **Figure 4C** and **Table 4**. In males, the cutoff value for TG/HDL-C was 4.08, whereas in females, it was 2.14. The difference between the two sexes is substantial, with the cutoff value in males being nearly twice that of females.

**Table 4:** AUC and cut-off values of IR surrogates for prediction of HUA.

Variables	Cut-off	AUC (95%*CI*)	Sensitivity (%)	Specificity (%)
Total				
TyG	8.98	0.696 (0.638, 0.754)	55.60	76.60
TyG-BMI	186.52	0.686 (0.626, 0.745)	84.80	21.70
TG/HDL-C	0.85	0.675 (0.618, 0.733)	100.0	8.90
METS-IR	32.21	0.674 (0.613, 0.734)	75.80	26.10
Male				
TyG	8.98	0.724 (0.643, 0.806)	61.20	78.90
TyG-BMI	205.10	0.661 (0.570, 0.752)	67.30	62.40
TG/HDL-C	4.08	0.701 (0.619, 0.783)	46.90	87.30
METS-IR	36.46	0.638 (0.547, 0.729)	49.00	76.10
Female				
TyG	8.76	0.678 (0.597, 0.759)	68.00	63.80
TyG-BMI	216.56	0.718 (0.640, 0.795)	72.00	69.50
TG/HDL-C	2.14	0.651 (0.569, 0.733)	68.00	58.90
METS-IR	38.48	0.707 (0.626, 0.788)	58.00	81.00

Only *P*<0.05 was shown.

**Figure 5:**
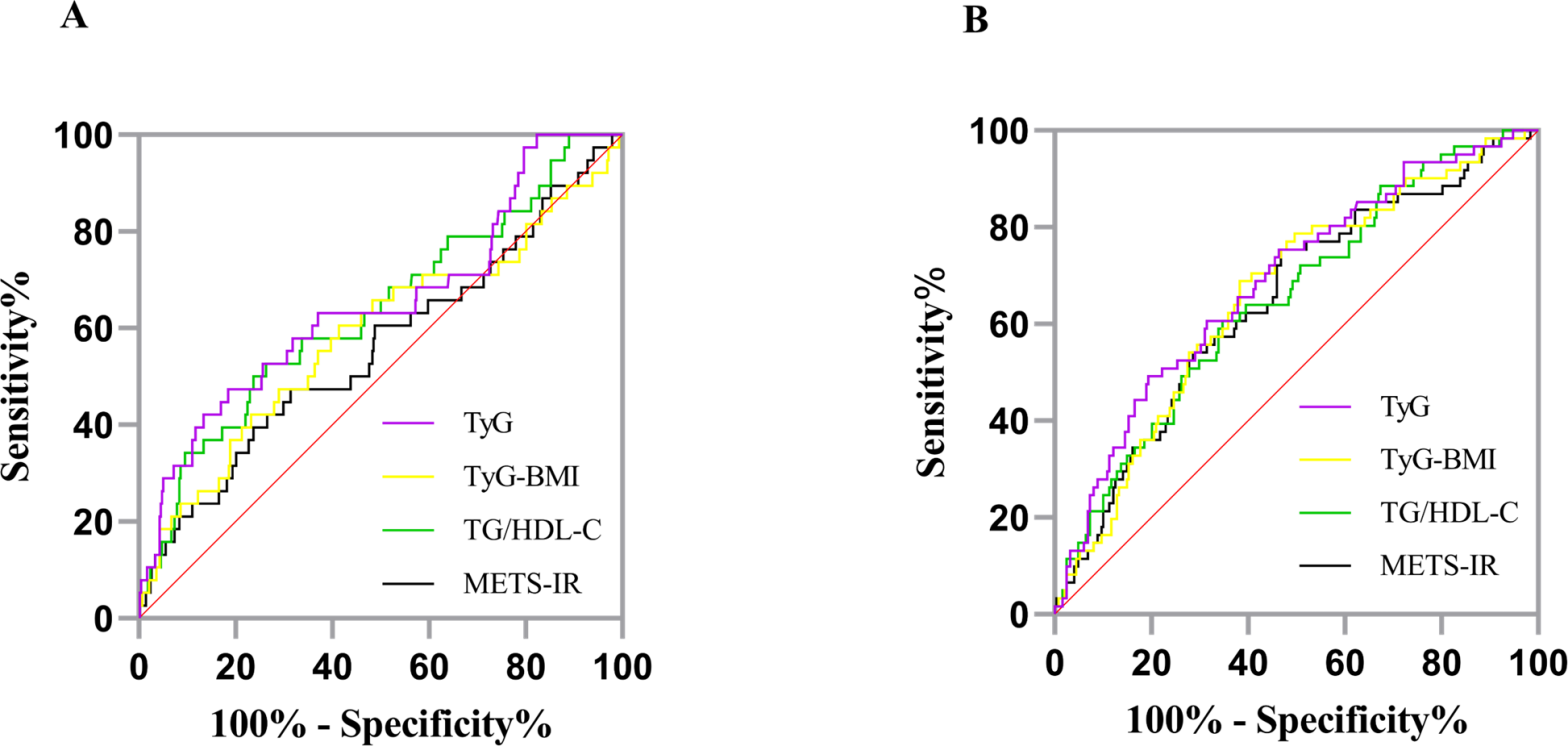
ROC curves for predicting HUA using different IR surrogate markers. **(A)** BMI < 24kg/m^2^; **(B)** BMI ≥ 24 kg/m^2^. HUA, hyperuricemia; TyG, triglyceride-glucose index; TyG-BMI, triglyceride-glucose with body mass index; TG/HDL-C, the ratio of triglyceride level divided by the high-density lipoprotein cholesterol level; METS-IR, the metabolic score for insulin resistance. Sensitivity, probability of HUA detection; 100% - Specificity%, probability of false alarm.

In this study, the predictive ability of some IR surrogates for HUA differed significantly between the BMI < 24 kg/m^2^ and BMI ≥ 24 kg/m^2^ groups. Only TyG and TG/HDL-C showed statistically significant differences (*P* < 0.05), and the AUCs of these indices were 0.650 and 0.635, respectively, indicating that both of these IR surrogates had certain predictive ability for HUA. TyG had the best predictive ability, and the optimal cutoff value of TyG was 8.98, as shown in **Figure 5A** and **Table 5**. In the group with BMI ≥ 24 kg/m^2^, the AUCs of TyG, TyG-BMI, TG/HDL-C, and METS-IR are 0.686, 0.660, 0.652, and 0.647, respectively. The optimal cutoff value of TyG was 9.22, which is shown in **Figure 5B** and **Table 5**.

**Table 5:** AUC and cut-off values of IR surrogates for prediction of HUA in different BMI groups.

Variables	Cut-off	AUC (95%CI)	Sensitivity (%)	Specificity (%)
BMI<24 (kg/m^2^)				
TyG	8.98	0.650 (0.550, 0.751)	47.40	81.60
TyG-BMI	–	–	–	–
TG/HDL-C	-0.61	0.635 (0.536, 0.734)	100.00	0.00
METS-IR	–	–	–	–
BMI≥24 (kg/m^2^)				
TyG	9.22	0.686 (0.612, 0.760)	49.20	80.60
TyG-BMI	239.23	0.660 (0.586, 0.734)	68.90	61.70
TG/HDL-C	-0.36	0.652 (0.576, 0.727)	100.00	0.00
METS-IR	38.54	0.647 (0.570, 0.723)	75.40	53.20

Only *P*<0.05 was shown.

## 4 Discussion

HUA exhibits a variable prevalence across Chinese populations in different regions of China, ranging from 2.6% to 36%; a national pooled prevalence is 13.3% (35). Recent surveys (2015-2019) indicate a rising trend, with the overall prevalence reaching 14.0% and affecting younger demographics (31). HUA prevalence among diabetic patients has been reported to be 21.24% in China (41), 10.7%- 45% in African (16), and 28.1% in Japanese (17). In this study, in 99 residents with HUA, diabetes did not develop suggesting HUA might not be the causative factor for diabetes, but IR/hyperinsulinemia could be the causative factor for HUA because in hyperinsulinemia more urate is reabsorbed in the proximal tubule via urate reabsorption transporters like the most crucial urate transporter, GLUT9 and also OAT10 (15). With the increase in age, the prevalence of HUA is significantly higher in males than in females. Higher SU level in adult males could be associated with higher levels of testosterone and lower levels of sex hormone-binding globulin or lifestyles (preference for smoking, alcohol…….) (42, 43). Previous studies (44–46) have suggested that smoking, alcohol consumption, hypertension, hyperlipidemia, overweight, and obesity were all risk factors for HUA. Our findings showed that the prevalence of HUA in males was higher than that in females, and that the detection rate of these risk factors in males of all age groups was significantly different from that in females. Our study demonstrated a significantly higher prevalence of HUA in males compared to females within the 45–74 age range. The underlying mechanisms for this disparity may involve sex hormones, lifestyle, and genetic factors. Existing research suggests that androgens, which predominate in males, can inhibit renal uric acid excretion (47), leading to its accumulation. In contrast, premenopausal women benefit from estrogen’s ability to promote uric acid excretion (48), resulting in lower levels. This gender gap narrows after menopause as estrogen levels decline. Furthermore, males are more likely to have HUA-risk habits, such as consuming a high-purine diet and alcohol, which increase uric acid production. Additionally, factors like greater work-related stress and a higher prevalence of sedentary behavior among males contribute to obesity, a known risk factor for elevated uric acid (49–52). Therefore, comprehensive management of tobacco and alcohol intake, blood pressure, blood lipids, and weight in daily life requires more attention.

SU is the end product of purine metabolism in humans, great apes, or hominids like gorilla, chimpanzee, gibbon apes (53). Reactive oxygen species(ROS; e.g., H_2_O_2_) is prouduced by the catalytic activity of xanthine oxidoreductase in humans using molecular oxygen (54). A previous study (55) has shown that higher-than-normal level of SU is closely associated with IR. In recent years, the traditional ‘glucocentric’ view of insulin resistance has shifted to a ‘lipocentric’ perspective. Inflammation (through infiltration of macrophages and other immune cells) at the site of adipose tissue, liver, muscle, and pancreas was shown as a link between obesity, metabolic syndrome, and type 2 diabetes (56). ROS cause insulin resistance in the peripheral tissues by affecting various points in insulin receptor signal transduction, ultimately resulting in decreased GLUT4 translocation and expression of the GLUT4 transporter in the cellular membrane (57, 58). Mitochondria contribute to ROS levels in nutrient-rich environments and induce stress pathways in the cell (57, 59, 60). Obesity is a contributing factor to metabolic disturbances and contributes to oxidative stress propagation (61). Excess free fatty acid-induced increased oxidative stress was shown associated with mitochondrial fragmentation in differentiated C2C12 muscle cells and aberrant fission in the skeletal muscle of obese mice (62); and elevated levels of SU can promote these two mechanisms to induce IR in various tissues and cells, such as pancreatic beta cells, fat cells, cardiomyocytes, vascular endothelial cells, macrophages, skeletal muscle, and liver. Adipose tissue IR is positively correlated with SU and HUA, but not with BMI grade. When BMI was in the normal range, the relationship between adipose tissue IR and SU was closer than that of liver IR (56). A bidirectional Mendelian randomization analysis (63) showed a positive causal relationship between IR and HUA, with hyperinsulinemia in the IR state leading to HUA. Studies have also shown that exogenous insulin reduces renal excretion of SU, thereby increasing the risk of HUA (64). Under conditions of hyperinsulinemia, more urate is reabsorbed (64–69)by the urate reabsorption transporters (especially by GLUT9 and OAT10) (15) in proximal tubule cells, resulting in HUA in the absence of renal dysfunction because proximal tubule cells retain their sensitivity to insulin in the state of insulin resistance (70). Insulin resistance leads to impaired UA excretion at a low urinary pH, contributing to the formation of urate stones (19).

In our study of non-diabetic individuals, no significant correlation was found between LDL-C and HUA, a significant inverse association was observed between HDL-C and HUA (**Table 1**). In contrast, HUA was previously reported as a risk for hypertriglyceridemia and high LDL-C but not for low HDL-C (71) or high HDL-C (72). In future studies, we will enroll a larger sample size and incorporate more comprehensive assessments to better control for potential confounding factors. Previously published reports showing association of the triglyceride glucose (TyG) index, TyG-body mass index (BMI), the ratio of triglycerides divided by high-density lipoprotein cholesterol (TG/HDL-C) and metabolic score for insulin resistance (METS-IR), TyG-waist-to-height ratio (WHtR) with the risk of HUA [SU = ≥ 420 umol/L (7 mg/dL)] by multiple studies in the general population in China and the United States (73), in patients with Nonalcoholic Fatty Liver Disease (NAFLD) (74), diabetic kidney disease (DKD) (75), patients with coronary heart disease (CHD) and hypertension (76, 77) or insulin resistance in Patients with Type 2 Diabetes (78). Our study also found that the AUCs of these four IR surrogates(TyG, TyG-BMI, TG/HDL-C, and METS-IR) for the total population were all >0.67, with the TyG showing the largest value (AUC = 0.696). Moreover, the best cutoff value was 8.98, as shown in **Figure 4A** and **Table 4**. For both male and female individuals, these four IR surrogates had good predictive ability for the occurrence of HUA in non-diabetic Yi residents in Yunnan and provided a reference for predicting the risk of HUA in the non-diabetic Yi residents. The AUC of the TyG in the male group was the largest (AUC = 0.724), and the best truncation value was 8.98, as shown in **Figure 4B** and **Table 4**. The AUC of the TyG-BMI in the female group was the largest (AUC = 0.718), and the best cutoff value was 216.56, as shown in **Figure 4C** and **Table 4**.

Since IR plays a certain role in the occurrence and development of HUA, rapid assessment of IR in high-risk groups is essential to identify the risk of HUA early. A large-sample study of middle-aged and elderly people with type 2 diabetes (26) showed that the TyG, TyG-BMI, TG/HDL-C, and METS-IR were independent influencing factors for the occurrence of HUA (*P* < 0.05); these four IR surrogates could predict the occurrence of HUA in middle-aged and elderly patients with type 2 diabetes. Another study (27) also indicated that among 7743 non-diabetic individuals in the United States, these four IR surrogates were closely related to HUA, the AUC of the TyG-BMI, and METS-IR in males or females were the largest (male: both AUC = 0.68; female: both AUC = 0.71). Thus, these findings showed that these four IR surrogates had good predictive ability for HUA in people with diabetes or not.

A study (28) suggested that in patients with coronary heart disease, the incidence of HUA in the highest quintile was 60% and 83%, higher than those in the first and second quintiles of the TyG and TyG-BMI. Another study (79) in a population in northeastern Iran showed that these four IR surrogates were significantly correlated with HUA, and the TyG-BMI had the best predictive effect. Studies have suggested that BMI can directly affect fasting insulin and HOMA−IR levels, and the correlation between them can be mediated by SU, while SU does not affect FPG (80). In our study, BMI and BMI ≥ 24 (kg/m^2^) were positively correlated with HUA. In the BMI group, the TyG had the highest predictive ability for HUA regardless of BMI classification, indicating that the TyG can be expected to be a screening tool for predicting a high risk of HUA in normal-BMI, overweight, or obese people. However, domestic studies (81, 82) have reported that the TyG-BMI and METS-IR have better sensitivity for identifying IR in overweight or obese people in comparison with the normal BMI group. Anyway the results of our study are mostly similar to those of the above researches (26, 27), with these four IR surrogates all showing some predictive ability for identifying the occurrence of HUA in non-diabetic Yi residents in Yunnan Province, but the IR surrogates with the best predictive ability in our study differed slightly across different subgroup analyses. These differences may be related to the ethnicity or the sample size. Subsequent studies in large sample populations encompassing different regions, races, ethnicities, and sexes may help validate these findings. In any case, these findings all confirmed the predictive ability of these four IR surrogates for HUA in non-diabetic Yi residents in Yunnan Province.

HUA is a metabolic syndrome caused by purine metabolism disorder in the human body, and is an independent risk factor for chronic kidney disease, hypertension, cardiovascular and cerebrovascular diseases, and diabetes (83). A retrospective cohort study of 3584 Japanese adults (84) showed that HUA in people with normal blood pressure was an independent risk factor for developing hypertension; in patients with prehypertension, the cumulative incidence of hypertension was significantly higher in men with SU levels above 7.0 mg/dL(≈420μmol/L) and in women with SU levels above 5.0 mg/dL(≈300μmol/L), and the risk of developing hypertension was significantly higher in women than in men. Another study (85) showed that in individuals with normal blood glucose levels, SU levels are associated with the incidence of hypertension only when blood pressure is less than 120/80 mmHg, while in middle-aged and elderly people, they are involved in the occurrence of hypertension in the absence of other risk factors.

A large cohort study (86) showed that patients with high SU levels had a significantly higher risk of unconscious vascular events than patients with low SU levels. Another study (87) has shown that the risk of mortality associated with coronary heart disease increases with every 1-mg/dL(≈60μmol/L) increase in SU (RR, 1.12; 95% *CI*, 1.05 to 1.19), and the risk was significantly higher in women than in men. A foreign study (88) showed a nonlinear relationship between SU level and mortality from chronic kidney disease, and patients with SU levels ≥ 5.900 mg/dL(≈354μmol/L) had a significantly higher risk of death from chronic kidney disease, but that study did not clarify whether the two showed a causal relationship. In addition, some studies (89) have shown that HUA can promote the death of pancreatic beta cells and lead to diabetes. A previous study (90) showed that in 212 women with normal blood pressure levels during pregnancy, a 1-mg/dL(≈60μmol/L) increase in the SU level was associated with a 1.23U increase in HOMA−IR (95% *CI*: 1.07-1.42; *p* = 0.003). Asymptomatic manifestations of HUA in the early clinical stage may lead to low attention and poor treatment compliance in patients with HUA, so asymptomatic high SU levels may develop into gout or cause damage to other target organs. Therefore, early identification and management of HUA and dynamic monitoring of its changes are of great significance.

Our study is the first to use the data of non-diabetic Yi residents in six areas of Yunnan to explore the relationship between these four IR surrogates and HUA, providing a simpler and more economical choice for clinical differentiation of IR status in non-diabetic HUA patients. However, the study had some limitations. For example, the sample size of this study is limited, and the findings may require validation in prospective studies with larger sample sizes.

## 5 Conclusions

Our study found that in non-diabetic Yi residents in six areas of Yunnan Province, the TyG, TyG-BMI, TG/HDL-C, and METS-IR were positively correlated with HUA. These four IR surrogates were good at predicting HUA. The TyG had the best predictive ability for the occurrence of HUA in non-diabetic Yi male residents, while the TyG-BMI had the best predictive ability for the occurrence of HUA in non-diabetic Yi female residents in six areas of Yunnan. In summary, our findings suggest that these four IR surrogates can be used as different reference surrogates to assess the risk of HUA in clinical and future epidemiological studies of the non-diabetic population. Among these four IR surrogates, the TyG and TyG-BMI have the best predictive ability and are simple and easy to obtain. Therefore, these surrogates can be expected to serve as reference indices for clinical practice, especially in primary medical institutions, to predict the occurrence of HUA.

## Data Availability

The original contributions presented in the study are included in the article/supplementary material. Further inquiries can be directed to the corresponding authors.
